# A Rare Case of Giant Inguinoscrotal Bladder Hernia as a Cause of Acute Respiratory Distress Syndrome

**DOI:** 10.7759/cureus.79518

**Published:** 2025-02-23

**Authors:** Thomas Neff, Caroline Amicone

**Affiliations:** 1 Intensive Care Unit, Centre Hospitalier Universitaire UCLouvain Namur, Godinne, BEL; 2 Intensive Care Unit, Centre Hospitalier Universitaire - Liège, Liège, BEL

**Keywords:** acute respiratory distress syndrome [ards], inguinal hernia, misdiagnosis, septic shock [ss], urinary bladder hernia

## Abstract

An inguinoscrotal hernia is a common condition in medical practice. However, an inguinoscrotal hernia involving the urinary bladder is a rare entity that can easily lead to diagnostic errors, resulting in inappropriate medical management. We report the case of a 77-year-old patient who presented to the emergency department with dysuria and testicular pain persisting for over a year. Clinical examination revealed a large, non-reducible inguinoscrotal hernia on palpation. Abdominopelvic computed tomography demonstrated a giant inguinoscrotal hernia containing almost the entire bladder, causing post-renal obstruction with upstream uretero-hydronephrosis. Emergency surgery was scheduled, involving inguinoscrotal hernia repair using the Lichtenstein technique. Postoperatively, the patient developed septic shock of urinary origin, complicated by severe acute respiratory distress syndrome (ARDS).

## Introduction

Inguinoscrotal hernia is a common condition in medical practice. However, a bladder-containing inguinoscrotal hernia is a rare entity that can easily lead to misdiagnosis, resulting in inappropriate medical management.

More than 90% of bladder hernias are discovered intraoperatively [1,2]. This diagnostic challenge is primarily due to the fact that most patients with bladder-containing inguinoscrotal hernias are asymptomatic [2]. Nevertheless, giant inguinoscrotal hernias are exceedingly rare and require surgical intervention, as they can lead to multiple complications, including renal failure, lithiasis within the herniated bladder, scrotal abscess, and even bladder necrosis [3-5].

Here, we report a case of a patient with an inguinoscrotal hernia involving nearly the entire bladder. The patient was initially misdiagnosed with an inguinal hernia, leading to life-threatening consequences.

## Case presentation

A 77-year-old patient presented to the emergency department with urinary complaints. He reported dysuria and testicular pain persisting for over a year, with worsening symptoms over the past week. Upon admission, the patient also exhibited dyspneic symptoms.

The patient had previously consulted his general practitioner and a urologist a few weeks earlier, who diagnosed a hydrocele. Clinical examination in the emergency department revealed an irreducible inguinoscrotal hernia on palpation. The abdomen was soft, non-distended, and non-tender upon examination.

**Figure 1:**
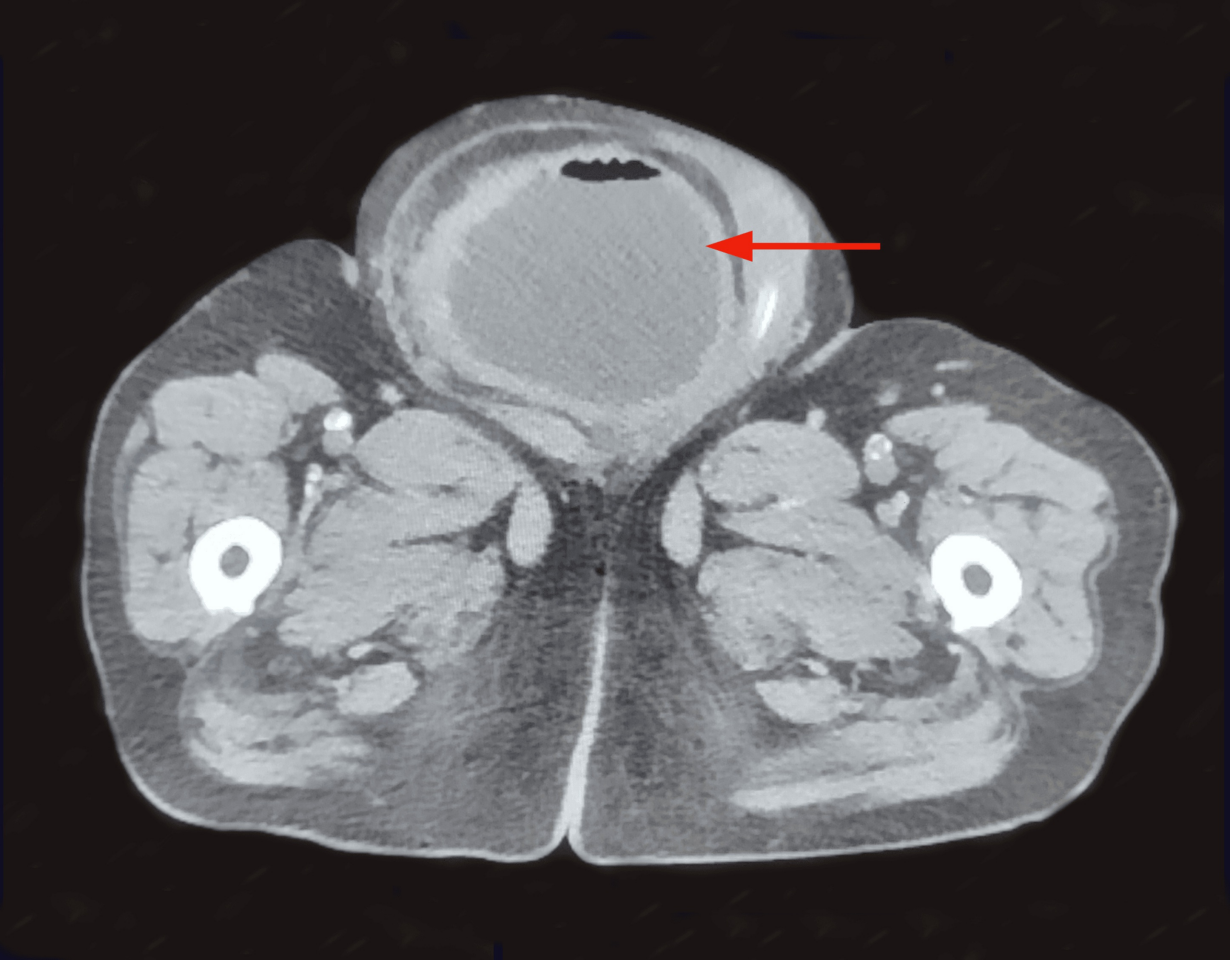
Transverse section of an abdominopelvic CT scan demonstrating a bladder-containing inguinoscrotal hernia (red arrow).

**Figure 2:**
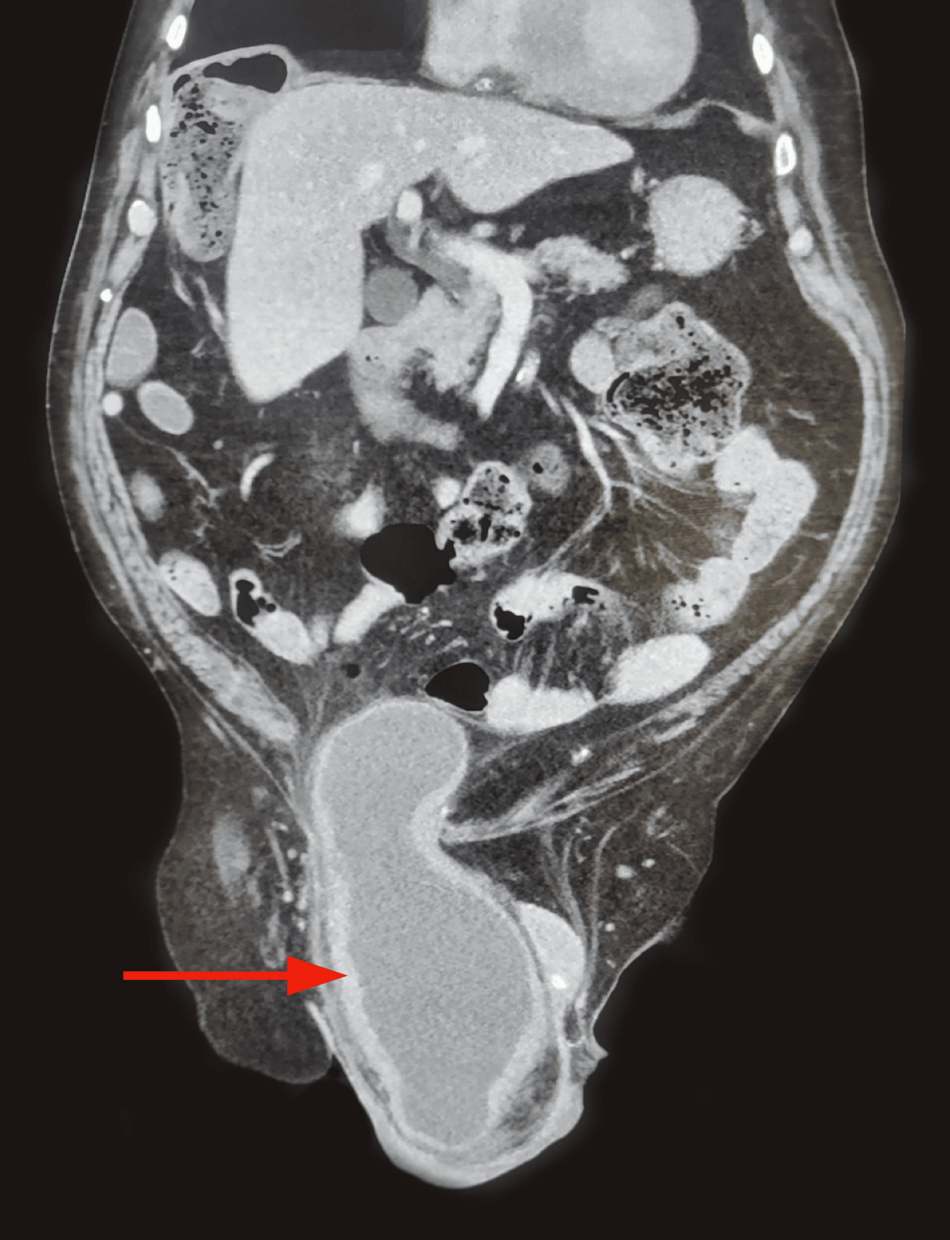
Sagittal section of an abdominopelvic CT scan demonstrating a bladder-containing inguinoscrotal hernia (red arrow).

Given the significant scrotal mass extending into the right inguinal region, an abdominopelvic CT scan was performed. The imaging revealed an inguinoscrotal hernia involving nearly the entire urinary bladder (Figures 1-2), resulting in a post-renal obstruction with upstream uretero-hydronephrosis.

The laboratory workup performed upon the patient's admission to the emergency department revealed a significant inflammatory syndrome, characterized by a marked elevation of C-reactive protein (CRP) and leukocytosis, indicating an underlying inflammatory or infectious process (Table 1). Concurrently, the patient exhibited acute kidney injury (AKI) classified as KDIGO stage II (Kidney Disease: Improving Global Outcomes), with an initial serum creatinine level of 1.45 mg/dL, which progressively worsened to 2.74 mg/dL after three days of hospitalization. The etiology of this AKI was attributed to a post-renal obstruction, leading to urinary stasis and bilateral uretero-hydronephrosis upstream.

**Table 1:** Laboratory results at the patient's admission to the emergency department.

Test	Value	Unit	Reference ranges
Hemoglobin	12.2	g/dL	13 - 16.5 g/dL
CRP	206.66	mg/L	< 8 mg/L
Leukocytes	14.74 x 10^3^	/uL	4 - 11 x 10^3^/uL
Creatinine	1.45	mg/dL	0.66 - 1.25 mg/dl
Glomerular filtration rate (GFR)	43.00	mL/min/1.73 m^2^	60 - 110.63 ml/min/1.73m^2^

Upon admission to the emergency department, the patient also presented with acute respiratory distress characterized by hypoxemia, requiring oxygen therapy via a high-concentration reservoir mask at a flow rate of up to 12 L/min. Pulmonary auscultation revealed diffusely diminished breath sounds, without wheezing or significant crackles. This respiratory failure was associated with a state of shock, with signs of peripheral hypoperfusion, including cutaneous mottling. The initial hemodynamic assessment showed persistent arterial hypotension despite initial fluid resuscitation with crystalloids, warranting the initiation of vasopressor support with norepinephrine.

From an infectious standpoint, the patient was febrile, with documented pyrexia (38.6°C) and a marked inflammatory response. Given the clinical and biological context, the leading diagnostic hypothesis was septic shock of urinary origin. Empirical intravenous antibiotic therapy with amoxicillin-clavulanic acid was promptly initiated. Regarding the bacteriological analysis, *Escherichia coli* was identified in the cytobacteriological examination of urine in the days following admission. However, blood cultures and expectorations samples failed to identify any organisms.

In light of the clinical presentation, an emergency surgical intervention was scheduled, consisting of an inguinoscrotal hernia repair using the Lichtenstein technique with the placement of a prosthetic mesh. The decision to place this prosthetic mesh was made by the visceral surgeon during the procedure. This decision was justified by the absence of necrotic-appearing tissue, as all tissues appeared healthy during the surgery. Additionally, there was no suspicion of perforation, either on imaging or during the surgical procedure.

From an anesthetic perspective, the procedure was notable for worsening gas exchange following intubation, necessitating the administration of 100% inspired oxygen, increased positive end-expiratory pressure, and multiple alveolar recruitment maneuvers. The surgery was also marked by progressive hemodynamic instability, requiring escalating norepinephrine doses, peaking at 0.8 µg/kg/min.

**Figure 3:**
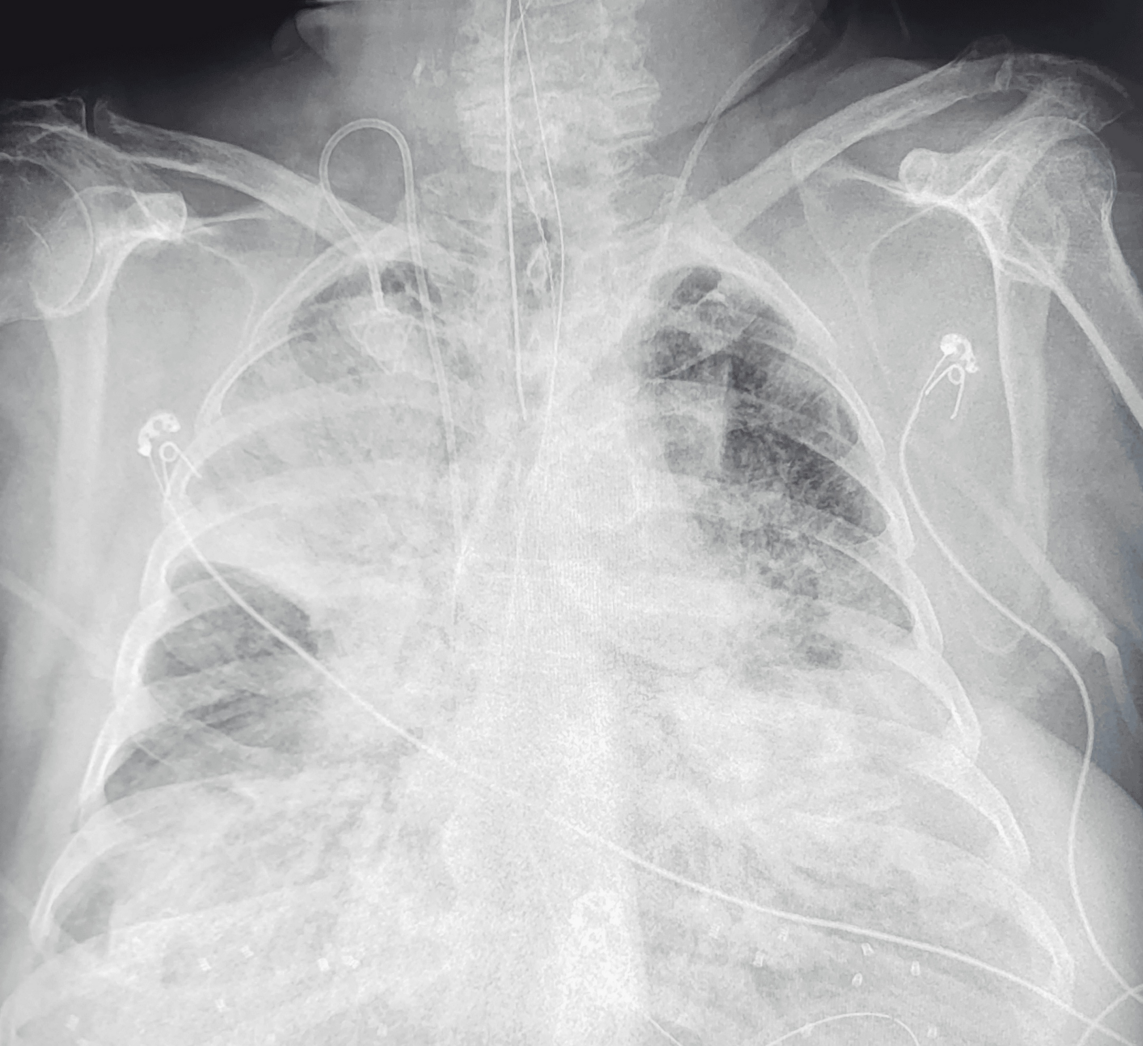
Chest X-ray demonstrating bilateral diffuse infectious pneumonia.

In this context, the patient was admitted to the intensive care unit (ICU) for postoperative management. A chest X-ray performed upon ICU admission revealed bilateral diffuse opacities (Figure 3). This radiological finding, in conjunction with the patient's clinical context, is suggestive of acute respiratory distress syndrome (ARDS) of urinary septic shock origin.

The patient was promptly placed in the prone position due to persistent hypoxemia following the surgical procedure (arterial oxygen pressure-to-inspired oxygen fraction ratio of 85).

The patient underwent prone positioning sessions in the days following admission to the intensive care unit. Additionally, he developed a type 2 myocardial infarction secondary to hypoxemia, with a peak troponin level of 27,960 ng/L (creatine phosphokinase at 895 IU/L) on the second day of hospitalization.

The patient was successfully extubated after seven days of invasive mechanical ventilation. However, post-extubation, he developed airway secretion retention, leading to a progressive deterioration in gas exchange. He was managed with alternating high-flow nasal oxygen therapy and non-invasive mechanical ventilation. Due to worsening dyspnea and increased airway secretions, reintubation was indicated four days after extubation. However, the patient expressed his wish to discontinue further treatment and refused reintubation. In this context, following a collegial decision, therapeutic de-escalation was initiated. The patient ultimately passed away after 11 days of hospitalization.

## Discussion

Bladder-containing inguinoscrotal hernia is an extremely rare clinical entity, accounting for only 0.5% to 3% of inguinal hernias [6]. This condition poses significant diagnostic and therapeutic challenges due to its nonspecific clinical presentation and potential for severe complications. In this case, an initial misdiagnosis led to a critical delay in treatment, ultimately resulting in septic shock and acute respiratory distress syndrome (ARDS), a previously unreported complication in the literature. Complications of bladder hernia are primarily related to poor bladder emptying, which can result in recurrent urinary tract infections, vesicoureteral reflux, bladder rupture, uretero-hydronephrosis, strangulation, and bladder ischemia. The clinical history should include urinary symptoms such as dysuria, urgency, and frequency of urination [7]. In advanced cases, two-stage voiding may occur, where spontaneous voiding is followed by manual compression of the herniated bladder or a noticeable reduction in scrotal size after urination [8]. The diagnosis of bladder-containing inguinoscrotal hernia remains challenging, with fewer than 7% of cases identified preoperatively and 16% only diagnosed postoperatively [1,2]. It should be considered in men over 50 years of age presenting with obstructive or irritative urinary symptoms [9]. The incidence of inguinal hernia increases with age, typically affecting men between 50 and 70 years [7,8]. Several risk factors contribute to bladder hernia development, including chronic urinary obstruction, obesity, advanced age, decreased bladder tone, and weakened pelvic musculature [1,3].

The treatment of bladder-containing inguinoscrotal hernia is invariably surgical, requiring bladder repositioning, inguinal hernia repair, and management of any subvesical obstruction. In this case, the patient was initially diagnosed with a hydrocele, leading to a delay in appropriate care. This misdiagnosis resulted in disease progression to septic shock, complicated by severe ARDS requiring invasive mechanical ventilation. While bladder hernia has been associated with pulmonary edema due to volume overload from obstructive uropathy [10], its direct link to ARDS has not been previously documented. Delayed diagnosis and inadequate initial management can lead to life-threatening complications. Several cases have also highlighted severe complications arising from untreated bladder-containing inguinoscrotal hernia due to diagnostic delays [3,10,11]. While inguinal hernia is typically a clinical diagnosis, imaging studies may be necessary in certain cases. Computed tomography (CT) imaging is particularly recommended for high-risk patients, such as men over 50 years old, obese individuals with urological symptoms or other causes of urinary outflow obstruction, recurrent lower urinary tract infections, reduced urinary flow, or diagnostic uncertainty [3,8,12]. The severe complications in our patient could have been avoided if the initial symptoms had been approached with greater vigilance. A thorough evaluation could have enabled an accurate diagnosis and early surgical management of the bladder-containing inguinoscrotal hernia, potentially preventing the patient’s progression to critical illness and its associated complications. A 2004 review of 190 cases of inguinal hernias with urological involvement found that 11.2% were associated with urological malignancies, while 23.5% resulted in complications such as renal failure and recurrent urinary infections [9]. This highlights the importance of confirming a bladder hernia diagnosis before surgical intervention. Preoperative identification of the hernia's contents is essential for avoiding surgical bladder injury and optimizing inguinal hernia repair [7]. Preoperative identification of the hernia's contents is crucial to avoiding surgical bladder injury and optimizing the operative management of inguinal hernia repair [7].

The novelty of this case lies in the unique progression to ARDS, highlighting the need for heightened clinical vigilance and the importance of early abdominal CT imaging in patients presenting with atypical inguinal or scrotal swelling. This case underscores the critical role of prompt diagnosis in preventing severe complications and contributes to the growing body of evidence advocating for a thorough evaluation of inguinoscrotal hernias that may be at risk of containing bladder tissue.

## Conclusions

This case report underscores the critical consequences of underestimating a medico-surgical condition and highlights the importance of early and accurate diagnosis.

The diagnosis of an inguinoscrotal bladder hernia is challenging due to its nonspecific clinical presentation, which may lead to misdiagnosis and inappropriate management. However, timely and precise identification is crucial, as this condition invariably requires surgical intervention. Delayed treatment can result in severe complications, potentially leading to life-threatening consequences.

We identified the risk factors associated with the development of a bladder-containing inguinoscrotal hernia and emphasized the essential role of imaging techniques in cases of diagnostic uncertainty.

Our patient, presenting with a bladder-containing inguinoscrotal hernia, was mistakenly diagnosed with a hydrocele. This diagnostic error led to a failure to recognize the need for timely surgical intervention. The delay in surgical management led to life-threatening consequences, including septic shock and ARDS, necessitating intensive care admission and ultimately resulting in death due to refractory hypoxemia.
